# Suppurative Nephritis in a Young Dromedary

**Published:** 1882-07

**Authors:** 


### III.—SUPPURATIVE NEPHRITIS IN A YOUNG DROMEDARY.

In December last a young dromedary arrived at this port from Hamburg, having been very much exposed to the unusually stormy weather of that month. During a voyage of twenty-three days the decks of the steamer were almost constantly washed by the heavy seas, and as the animal was part of the deck load, he was wet a good deal of the time. On his arrival great weakness was manifested, in fact, he could hardly stand. No other symptoms were noticed. On the second day after arrival at the Central Park menagerie he died. At post mortem examination hypostatic œdema of the lungs was found, and, in conjunction, suppurative nephritis, the kidneys being the seat of abscesses, and their substances gangrenous. This acute attack was presumed to have been induced by the constant exposure on deck during the ocean voyage.

CENTRAL PARK, NEW YORK CITY.

